# Effectiveness of 2023 southern hemisphere influenza vaccines against severe influenza-associated illness: pooled estimates from eight countries using the test-negative design

**DOI:** 10.1016/S2214-109X(24)00473-X

**Published:** 2025-01-29

**Authors:** Radhika Gharpure, Annette K Regan, Francisco Nogareda, Allen C Cheng, Christopher C Blyth, Siobhan St George, Q Sue Huang, Tim Wood, Andrew Anglemyer, Kriengkrai Prasert, Prabda Praphasiri, William W Davis, Chakrarat Pittayawonganon, Regina Ercole, Analia Iturra, Walquiria Aparecida Ferreira de Almeida, Francisco José de Paula Júnior, Marcela Avendaño Vigueras, Maria Fernanda Olivares Barraza, Chavely Domínguez, Elena Penayo, Natalia Goñi, Daiana Tritten, Paula Couto, Daniel Salas, Ashley L Fowlkes, Lindsey M Duca, Eduardo Azziz-Baumgartner, Sheena G Sullivan

**Affiliations:** a US Centers for Disease Control and Prevention, Atlanta, GA, USA; b Pan American Health Organization, Washington, DC, USA; c Fielding School of Public Health, University of California Los Angeles, Los Angeles, CA, USA; d School of Nursing and Health Professions, University of San Francisco, San Francisco, CA, USA; e Monash Infectious Diseases, Monash Health and School of Clinical Sciences, Monash University, Melbourne, VIC, Australia; f School of Medicine and Wesfarmers Centre of Vaccines and Infectious Disease, Telethon Kids Institute, University of Western Australia, Nedlands, WA, Australia; g Department of Infectious Diseases, Perth Children's Hospital, Nedlands, WA, Australia; h PathWest Laboratory Medicine WA, Queen Elizabeth II Medical Centre, Perth, WA, Australia; i Interim Australian Centre for Disease Control, Canberra, ACT, Australia; j Institute of Environmental Science and Research, Wellington, New Zealand; k Nakhon Phanom Hospital, Ministry of Public Health, Nonthaburi, Thailand; l Department of Disease Control, Ministry of Public Health, Nonthaburi, Thailand; m Faculty of Public Health, Kasetsart University, Sakon Nakhon, Thailand; n Thailand Ministry of Public Health–US CDC Collaboration, Nonthaburi, Thailand; o Unidad centinela de IRAG, Hospital San Juan de Dios, Buenos Aires, Argentina; p Unidad centinela de IRAG, Hospital Zonal de Trelew “Dr Adolfo Margara”, Chubut, Argentina; q Ministry of Health, Brasilia, Brazil; r Departamento de Inmunizaciones, Ministerio de Salud, Santiago, Chile; s Departamento de Epidemiologia, Ministerio de Salud, Santiago, Chile; t General Directorate of Health Surveillance, Ministry of Public Health and Social Welfare, Asunción, Paraguay; u Department of Public Health Laboratories, Ministry of Health, Montevideo, Uruguay; v Department of Health Surveillance, Ministry of Health, Montevideo, Uruguay

## Abstract

**Background:**

Annual estimates of seasonal influenza vaccine effectiveness can guide global risk communication and vaccination strategies to mitigate influenza-associated illness. We aimed to evaluate vaccine effectiveness in countries using the 2023 southern hemisphere influenza vaccine formulation.

**Methods:**

We evaluated end-of-season influenza vaccine effectiveness across eight countries (Argentina, Australia, Brazil, Chile, New Zealand, Paraguay, Thailand, and Uruguay) that used the 2023 southern hemisphere vaccine formulation, with use of a test-negative design. All patients who attended participating hospitals with severe acute respiratory illness were tested by RT-PCR for influenza. We calculated country-specific, network-specific, and pooled vaccine effectiveness against hospitalisation. For countries with sufficient data, we also calculated vaccine effectiveness against intensive care unit (ICU) admission by comparing the odds of vaccination among test-positive cases to that among test-negative controls. We evaluated vaccine effectiveness for groups prioritised for vaccination (young children aged 1–4 years, people aged 5–64 years with underlying health conditions, and older adults aged ≥65 years).

**Findings:**

From March 5 to Nov 27, 2023, 31 368 individuals were admitted to hospital with severe acute respiratory infection in the eight included countries. Of these, 12 609 individuals admitted to hospital (6452 [51·2%] female and 6157 [48·8%] male) who met inclusion criteria and had complete data were included in the analysis, including 4388 test-positive cases and 8221 test-negative controls. Pooled vaccine effectiveness against hospitalisation with any influenza virus was 51·9% (95% CI 37·2–66·7), with substantial heterogeneity across countries (*I*^2^ 74%). Vaccine effectiveness against ICU admission from any influenza virus was 67·7% (44·5–81·2) in Chile and 69·7% (45·3–83·3) in Australia. Vaccine effectiveness estimates against hospitalisation were highest for young children (70·9% [47·5–94·4]) and lowest for older adults (47·7% [24·9–70·5]).

**Interpretation:**

Across eight countries, 2023 southern hemisphere vaccines were effective in reducing hospitalisations from influenza illness. Use of common protocols can facilitate data pooling to provide a comprehensive evaluation of vaccine effectiveness across settings.

**Funding:**

US Centers for Disease Control and Prevention cooperative agreements to the Pan American Health Organization and the Thailand Ministry of Public Health; the Australian Government Department of Health and Aged Care; and the New Zealand Ministry of Health.

## Introduction

Annual estimates of influenza vaccine effectiveness provide valuable evidence about the effect of vaccination programmes in reducing illness from influenza virus infection, thereby guiding efforts to minimise the burden of seasonal influenza on health-care systems and communities. Public health authorities can use vaccine effectiveness estimates to tailor risk communication strategies about the importance of influenza vaccination as a preventive measure, particularly for populations at higher risk of developing severe complications from influenza virus infection, such as young children, older adults, and people with underlying health conditions.^1^ Additionally, national and regional vaccine effectiveness estimates are shared with WHO each February and September to contribute to the global influenza vaccine strain selection.^2^ Experts use data on southern hemisphere virus circulation and vaccine effectiveness data to inform northern hemisphere strain selection, and vice versa.^2^, ^3^

**Research in context**

**Evidence before this study**

Each February, countries using the southern hemisphere formulation of the seasonal influenza vaccine share vaccine effectiveness estimates with WHO to inform vaccine composition decisions, although this report is not publicly available. In March, 2024, we performed an informal literature review in PubMed for articles containing the terms “severe acute respiratory illness” and “2023” and “southern hemisphere” and “vaccine effectiveness”, and did not identify any published studies. We identified one publication that presented mid-season 2023 vaccine effectiveness from REVELAC-i (Network for the Evaluation of Influenza Vaccine Effectiveness in Latin America and the Caribbean) in South American countries.

**Added value of this study**

In this analysis, we evaluated 2023 end-of-season influenza vaccine effectiveness against hospitalisation across eight countries (Argentina, Australia, Brazil, Chile, New Zealand, Paraguay, Thailand, and Uruguay), encompassing 12 609 hospitalised individuals with severe acute respiratory illness. Our findings indicate that 2023 southern hemisphere vaccines were effective in reducing influenza-associated hospitalisation. In countries with sufficient data, we also found that the vaccines were effective in reducing influenza-associated intensive care unit admission.

**Implications of all the available evidence**

The 2023 southern hemisphere influenza vaccines were effective in reducing influenza-associated severe outcomes. These multiregion, multicountry vaccine effectiveness estimates can facilitate risk communication during the northern hemisphere season, inform vaccine composition decisions, and inform on vaccine effectiveness for outcomes and among subgroups constrained by sample size.

To generate annual estimates of influenza vaccine effectiveness against hospitalisation, many countries use data collected through sentinel surveillance for test-negative design analyses.^4^, ^5^ Single-country vaccine effectiveness estimates can be limited by small sample size and might vary because of geographical heterogeneity in virus circulation;^6^ thus, examination of data from multiple countries and use of common protocols to pool data can facilitate vaccine effectiveness estimation for a given season. In this report, we evaluate 2023 end-of-season influenza vaccine effectiveness against hospitalisation across eight countries (Argentina, Australia, Brazil, Chile, New Zealand, Paraguay, Thailand, and Uruguay) that used the 2023 southern hemisphere vaccine formulation.^7^ We also assess the effectiveness of influenza vaccination against intensive care unit (ICU) admission, against specific influenza subtypes, and for specific groups prioritised for influenza vaccination by the WHO Strategic Advisory Group for Immunization (ie, young children aged 1–4 years, children and adults aged 5–64 years with underlying health conditions, and older adults aged ≥65 years).^1^

## Methods

### Data sources and study design

We describe the surveillance systems and networks used in this analysis in the appendix (pp 3–5). For Australia, surveillance data were obtained from the Influenza Complications Alert Network (FluCAN)—a network of 21 sentinel hospitals located nationwide.^8^ For Thailand, data were obtained from nine hospitals conducting severe acute respiratory infection (SARI) surveillance across eight provinces.^9^ For New Zealand, data were obtained from four hospitals in Auckland and Counties Manukau District Health Boards.^10^ For Argentina, Brazil, Chile, Paraguay, and Uruguay, data were obtained from REVELAC-i (Network for the Evaluation of Influenza Vaccine Effectiveness in Latin America and the Caribbean)—a multicountry SARI sentinel surveillance-based network established by the Pan American Health Organization (PAHO) to estimate influenza vaccine effectiveness in Latin America and the Caribbean. The data represented 11 sentinel hospitals in Argentina, 455 in Brazil, eight in Chile, two in Paraguay, and ten in Uruguay.^11^, ^12^ Most countries used a standardised SARI case definition for surveillance, defined as an acute respiratory infection with history of fever or measured fever of 38°C or higher and cough, with onset within the past 10 days and requiring hospitalisation;^13^ Australia used a modified case definition (appendix pp 3–5).

The vaccine effectiveness evaluation start date for each country was the date of illness onset (or date of hospital admission, if illness onset was not reported) of the first hospitalised case with RT-PCR-confirmed influenza occurring after the start of the country's 2023 seasonal influenza vaccination campaign and the start of the 2023 influenza season (appendix pp 3–5); the evaluation end date was the date of onset of the last reported hospitalisation up to the end of the country's 2023 influenza season, or Nov 30, 2023, if no clear seasonality was observed. Influenza seasonality was determined from examination of weekly influenza virus detections for each country.

Countries shared de-identified line-list data for this multicountry analysis, including: patient demographics; underlying medical conditions as defined in the appendix (pp 3–5); influenza vaccination history; dates of illness onset, hospital admission, and sample collection; RT-PCR test results; type and subtype of influenza virus detected; and clinical outcomes. Influenza vaccine formulation was not reported; we assumed that all patients received the southern hemisphere formulation offered in the participating countries. We defined cases as patients admitted to hospital with SARI and a positive RT-PCR result for influenza virus; controls were patients admitted to hospital with SARI and negative RT-PCR results for both influenza virus and SARS-CoV-2.^14^ If the date of illness onset was not reported, it was imputed with the reported hospital admission date. We classified patients as vaccinated if they received at least one dose of the 2023 season influenza vaccine at least 14 days before illness onset or hospital admission; patients who did not receive any dose of the 2023 season influenza vaccine at the time of illness onset were classified as unvaccinated. Vaccination status was determined by means of vaccine registries or self report, depending on the country. We excluded any patients who: had a missing RT-PCR result for influenza; had an illness onset date before the start of the country's 2023 seasonal influenza vaccination campaign; had an illness onset date outside of the country's seasonal influenza activity; were younger than 1 year on the date of illness onset (many countries vaccinate children aged 6 months and older^1^ but this approach allowed for two doses of influenza vaccine before illness onset); had a sample collection date more than 10 days after the onset of symptoms; had a missing date of vaccination or a date of vaccination less than 14 days before illness onset; had a positive test result for SARS-CoV-2;^14^ or were missing information on vaccination status, underlying health conditions, sex, or age.

### Data analysis

For each country, we calculated vaccine effectiveness from the odds ratio (OR) comparing influenza vaccination among test-positive cases versus test-negative controls (vaccine effectiveness = [1 − OR] × 100%), using a logistic regression model adjusted for age group (1–4 years, 5–64 years, and ≥65 years), sex, underlying health conditions, and week of symptom onset (fit as a cubic spline). We additionally used multilevel mixed models to estimate a total vaccine effectiveness using data from all countries and within multicountry networks (ie, REVELAC-i). Mixed models fit a random intercept for country to account for potential country-level differences. We generated vaccine effectiveness estimates against SARI hospitalisation and ICU admission for each country by influenza virus subtype, and generated estimates for specific target groups (young children aged 1–4 years, children and adults aged 5–64 years with underlying health conditions, and older adults aged ≥65 years). For Brazil, young children included those aged 1–5 years, and older adults included those aged ≥60 years, consistent with the national influenza vaccination policy. We retained these age groupings for the primary analysis; however, for subgroup analyses by age group, we removed children aged 5 years and older adults aged 60–64 years from the Brazil data for consistency with programmes in other countries.

To evaluate consistency of vaccine effectiveness estimates across countries, we conducted random-effects meta-analyses of all countries with sufficient data to estimate vaccine effectiveness. Estimates were excluded if there were insufficient data (ie, cell with n<5 for 2 × 2 table comparing vaccination among test-positive cases *vs* test-negative controls) or if the 95% CI width exceeded 140%. Heterogeneity among country estimates was assessed using *I*^2^ and τ^2^ statistics.^15^ Pooled vaccine effectiveness estimates were only generated if three or more country-level estimates were available. All analyses were performed in Stata SE (version 17.0) and R (version 4.2.2).

### Ethical considerations

Surveillance systems and vaccine effectiveness networks were reviewed by in-country ethics review committees. In Australia, ethics approval was provided by the National Mutual Acceptance scheme at Alfred Health (51662-154/19), and in New Zealand, by the Northern A Health and Disability Ethics Committee (NTX/11/11/102). For REVELAC-i, the US Centers for Disease Control and Prevention (CDC) Public Health Ethics Committee, based on its review of the REVELAC-i protocol, ruled that the proposed multicentre evaluation was an evaluation programme and not a research project. Thailand sentinel surveillance did not require ethics review. Additionally, this pooled analysis was reviewed by the CDC, was deemed to not constitute human subjects research, and was conducted consistent with applicable federal law and CDC policy.

### Role of the funding source

Investigators from the funding agencies were involved in the study and contributed to the study design, the analysis and interpretation of data, the writing of the report, and the submission of results for publication. However, the funding agencies themselves were not involved in the interpretation of findings or the decision to submit for publication.

## Results

From March 5 to Nov 27, 2023, 31 368 individuals were admitted to hospital with SARI in Argentina, Australia, Brazil, Chile, New Zealand, Paraguay, Thailand, and Uruguay; of these, 18 262 (58·2%) were eligible for inclusion in the vaccine effectiveness analysis, 12 609 (69·0%) of whom had complete vaccination and covariate information (appendix p 6). The most common reasons for exclusion were that patients had a missing RT-PCR result for influenza (n=4787), tested positive for SARS-CoV-2 (n=2384), were vaccine ineligible (n=2206), or had a date of symptom onset before the introduction of the seasonal vaccine (exclusions were applied sequentially). We excluded 5653 patients with missing data on one or more of: vaccination status, pre-existing health conditions, sex, or age.

The 12 609 eligible patients with SARI (6452 [51·2%] female and 6157 [48·8%] male) included 4401 (34·9%) patients from Australia, 2782 (22·1%) from Chile, 2562 (20·3%) from Brazil, 934 (7·4%) from Uruguay, 857 (6·8%) from New Zealand, 734 (5·8%) from Thailand, 181 (1·4%) from Argentina, and 158 (1·3%) from Paraguay (appendix p 6). 4693 (37·2%) patients were young children aged 1–4 years, 4488 (35·6%) were children and adults aged 5–64 years, and 3428 (27·2%) were older adults aged 65 years or older (table). Among the total eligible population, 7075 (56·1%) patients had one or more reported underlying health conditions. Vaccination status ranged widely across countries, from 1428 (51·3%) of 2782 patients in Chile to 43 (5·9%) of 734 in Thailand.

**Table:** Patient characteristics for southern hemisphere influenza vaccine effectiveness estimation against severe acute respiratory infection hospitalisation in eight countries in 2023

			**Total**	**Australia**	**New Zealand**	**REVELAC-i countries**					**Thailand**
						Argentina	Brazil	Chile	Paraguay	Uruguay	
All patients			12 609	4401	857	181	2562	2782	158	934	734
Sex											
	Female		6452 (51·2%)	2297 (52·2%)	393 (45·9%)	81 (44·8%)	1274 (49·7%)	1461 (52·5%)	82 (51·9%)	452 (48·4%)	412 (56·1%)
	Male		6157 (48·8%)	2104 (47·8%)	464 (54·1%)	100 (55·2%)	1288 (50·3%)	1321 (47·5%)	76 (48·1%)	482 (51·6%)	322 (43·9%)
Group at risk											
	Young children (age 1–4 years)		4693 (37·2%)	1200 (27·3%)	267 (31·2%)	37 (20·4%)	1761 (68·7%)	595 (21·4%)	75 (47·5%)	348 (37·3%)	410 (55·9%)
	Children and adults (age 5–64 years) with underlying health conditions		4488 (35·6%)	2334 (53·0%)	375 (43·8%)	23 (12·7%)	2 (0·1%)	1194 (42·9%)	1 (0·6%)	293 (31·4%)	266 (36·2%)
	Older adults (age ≥65 years)		3428 (27·2%)	867 (19·7%)	215 (25·1%)	121 (66·9%)	799 (31·2%)	993 (35·7%)	82 (51·9%)	293 (31·4%)	58 (7·9%)
Underlying health condition											
	At least one condition		7075 (56·1%)	2696 (61·3%)	553 (64·5%)	129 (71·3%)	1184 (46·2%)	1788 (64·3%)	95 (60·1%)	450 (48·2%)	180 (24·5%)
	None		5534 (43·9%)	1705 (38·7%)	304 (35·5%)	52 (28·7%)	1378 (53·8%)	994 (35·7%)	63 (39·9%)	484 (51·8%)	554 (75·5%)
Influenza vaccination status											
	Vaccinated		3530 (28·0%)	921 (20·9%)	218 (25·4%)	27 (14·9%)	770 (30·1%)	1428 (51·3%)	21 (13·3%)	102 (10·9%)	43 (5·9%)
	Unvaccinated		9079 (72·0%)	3480 (79·1%)	639 (74·6%)	154 (85·1%)	1792 (69·9%)	1354 (48·7%)	137 (86·7%)	832 (89·1%)	691 (94·1%)
Influenza PCR result											
	Positive		4388 (34·8%)	2856 (64·9%)	207 (24·2%)	35 (19·3%)	509 (19·9%)	451 (16·2%)	44 (27·8%)	132 (14·1%)	154 (21·0%)
		Influenza A	3072 (24·4%)	1824 (41·4%)	147 (17·2%)	35 (19·3%)	409 (16·0%)	386 (13·9%)	40 (25·3%)	110 (11·8%)	121 (16·5%)
		Influenza A(H1N1)pdm09	2022 (16·0%)	1137 (25·8%)	35 (4·1%)	14 (7·7%)	233 (9·1%)	381 (13·7%)	40 (25·3%)	103 (11·0%)	79 (10·8%)
		Influenza A(H3N2)	167 (1·3%)	114 (2·6%)	14 (1·6%)	0	0	0	0	0	39 (5·3%)
		Influenza B	1305 (10·3%)	1020 (23·2%)	60 (7·0%)	0	100 (3·9%)	65 (2·3%)	4 (2·5%)	22 (2·4%)	34 (4·6%)
	Negative		8221 (65·2%)	1545 (35·1%)	650 (75·8%)	146 (80·7%)	2053 (80·1%)	2331 (83·8%)	114 (72·2%)	802 (85·9%)	580 (79·0%)

REVELAC-i=Network for the Evaluation of Influenza Vaccine Effectiveness in Latin America and the Caribbean.

**Figure 1:**
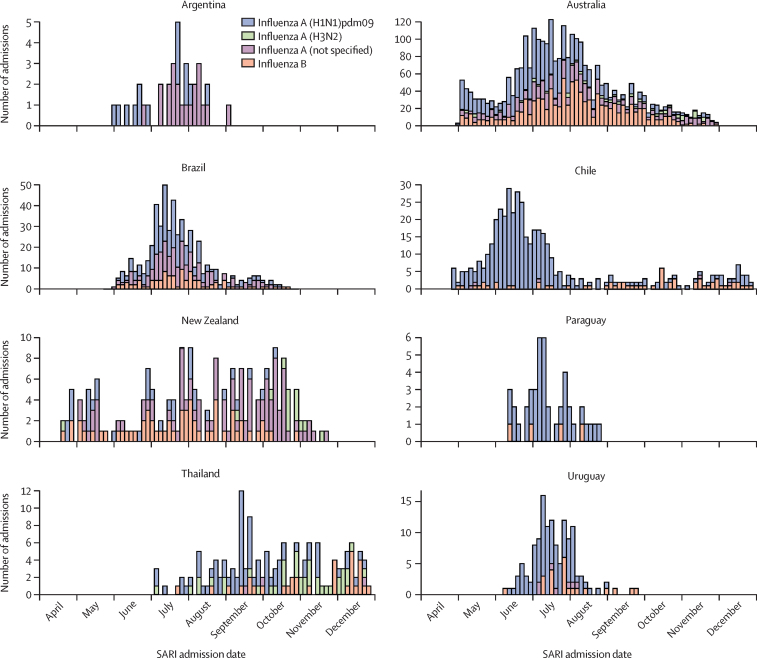
Influenza virus types and subtypes identified in patients admitted to hospital with SARI in eight countries in 2023 SARI=severe acute respiratory infection.

Among all patients, 4388 (34·8%) had a positive RT-PCR test for influenza (test-positive cases), and 8221 (65·2%) were negative for both influenza and SARS-CoV-2 (test-negative controls; table). Among cases, specimens from 3072 (70·0%) patients were positive for influenza A, of which 2022 (65·8%) were influenza A(H1N1)pdm09, 167 (5·4%) were influenza A(H3N2), and 883 (28·7%) were not subtyped; additionally, specimens from 1305 (29·7%) were positive for influenza B and 11 (0·3%) had no typing result. Of note, no influenza A(H3N2) was detected among REVELAC-i patients. Influenza season started in March–April and, for most countries, influenza detections peaked in May–July; however, circulation in New Zealand and Thailand did not show a distinct peak (figure 1).

**Figure 2:**
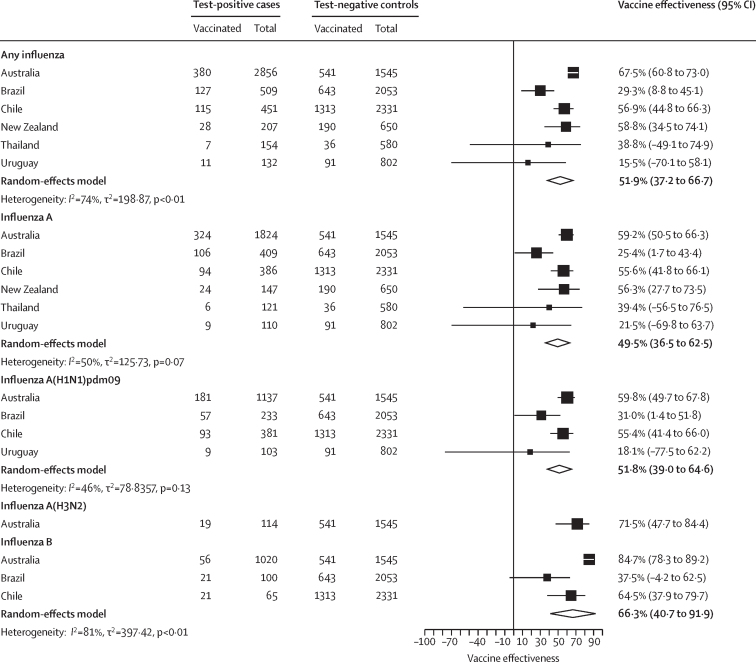
Estimated southern hemisphere influenza vaccine effectiveness against hospital admission for severe acute respiratory infection, by virus type and subtype, in 2023

Among all 4388 patients with a positive influenza test result, 672 (15·3%) had received the 2023 southern hemisphere influenza vaccine, compared with 2858 (34·8%) of the 8221 test-negative controls (appendix pp 7–8). Overall, the pooled vaccine effectiveness against SARI hospitalisation from any influenza virus was 51·9% (95% CI 37·2 to 66·7), with high heterogeneity across countries (*I*^2^ 74%; figure 2). Country-specific estimates (among countries with sufficient data) ranged from 67·5% (60·8 to 73·0) for Australia to 15·5% (–70·1 to 58·1) for Uruguay (figure 2). Across all participating South American countries in the REVELAC-i network, the pooled vaccine effectiveness estimate was 43·9% (33·8 to 52·5; appendix pp 7–8).

For influenza A, pooled vaccine effectiveness against SARI hospitalisation was 49·5% (95% CI 36·5 to 62·5) and heterogeneity was moderate (*I*^2^ 50%), with country-specific estimates ranging from 59·2% (50·5 to 66·3) to 21·5% (–69·8 to 63·7; figure 2; appendix pp 7–8). For influenza A(H1N1)pdm09, pooled vaccine effectiveness against SARI hospitalisation was 51·8% (39·0 to 64·6) with moderate heterogeneity (*I*^2^ 46%), with country-specific estimates ranging from 59·8% (49·7 to 67·8) to 18·1% (–77·5 to 62·2). For influenza A(H3N2), only Australia had sufficient test-positive patients to generate a country-specific estimate (71·5%; 95% CI 47·7 to 84·4). No influenza A(H3N2) detections were made in the participating REVELAC-i countries. For influenza B, pooled vaccine effectiveness against SARI hospitalisation was 66·3% (40·7 to 91·9) with substantial heterogeneity (*I*^2^ 81%), with country-specific estimates of 84·7% (78·3 to 89·2) for Australia, 64·5% (37·9 to 79·7) for Chile, and 37·5% (–4·2 to 62·5) for Brazil.

**Figure 3:**
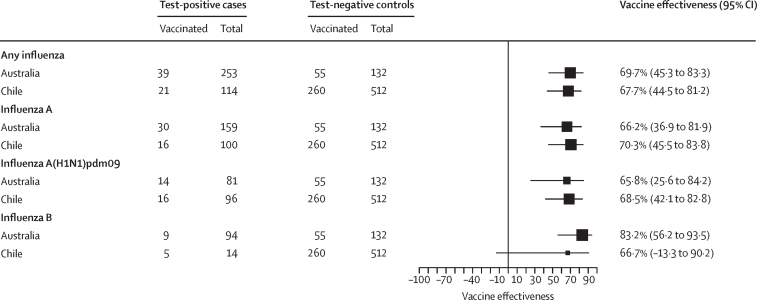
Estimated southern hemisphere influenza vaccine effectiveness against intensive care unit admission, by virus type and subtype, in 2023

Of the 12 609 patients, 1398 (11·1%) were reported to be admitted to an ICU, including 443 test-positive cases and 955 test-negative controls (appendix pp 9–10). Vaccine effectiveness against ICU admission from any influenza virus ranged from 69·7% (95% CI 45·3 to 83·3) in Australia to 67·7% (44·5 to 81·2) in Chile (figure 3; appendix pp 9–10). By type or subtype, vaccine effectiveness against ICU admission ranged from 70·3% (Chile) to 66·2% (Australia) for influenza A, from 68·5% (Chile) to 65·8% (Australia) for influenza A(H1N1)pdm09, and from 83·2% (Australia) to 66·7% (Chile) for influenza B. The numbers of ICU-admitted patients with influenza A(H3N2) infection were insufficient to yield a subtype-specific estimate.

**Figure 4:**
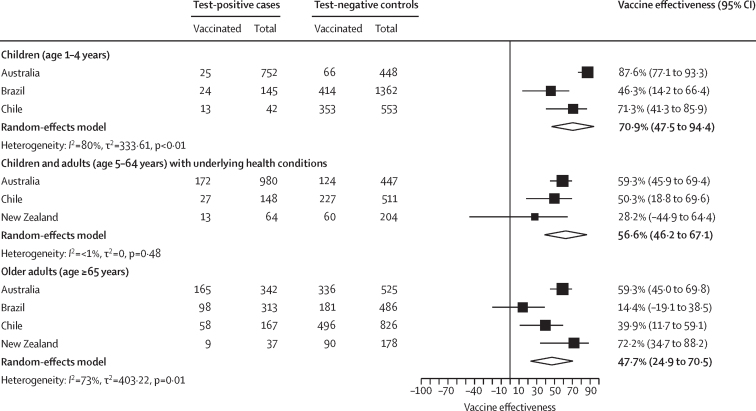
Estimated southern hemisphere influenza vaccine effectiveness against severe acute respiratory infection hospitalisation, by group at high risk, in 2023

For young children aged 1–4 years, pooled vaccine effectiveness against SARI hospitalisation was 70·9% (95% CI 47·5 to 94·4); heterogeneity was high (*I*^2^ 80%), with country-specific estimates ranging from 87·6% (77·1 to 93·3) for Australia to 46·3% (14·2 to 66·4) for Brazil (figure 4; appendix pp 11–14). For children and adults aged 5–64 years with underlying health conditions, pooled vaccine effectiveness against SARI hospitalisation was 56·6% (46·2–67·1); heterogeneity was low (*I*^2^ <1%) and country-specific estimates ranged from 59·3% (45·9–69·4) for Australia to 28·2% (–44·9 to 64·4) for New Zealand (figure 4; appendix pp 13–16). Lastly, for older adults aged ≥65 years, pooled vaccine effectiveness against SARI hospitalisation was 47·7% (24·9 to 70·5); heterogeneity was high (*I*^2^ 73%), with country-specific estimates ranging from 59·3% (45·0 to 69·8) for Australia to 14·4% (–19·1 to 38·5) for Brazil (figure 4; appendix pp 13–14, 17–18).

## Discussion

Our results indicate that vaccination with the 2023 southern hemisphere influenza vaccines reduced the odds of influenza-associated hospitalisations by approximately half across eight countries spanning Latin America, Asia, and Oceania (vaccine effectiveness 51·9% [95% CI 37·2–66·7]). These estimates were consistent with early-season 2023–24 northern hemisphere influenza vaccine effectiveness estimates against hospitalisation reported from the USA (52–61% in paediatric patients; 41–44% in adult patients),^16^ Canada (49–75%, depending on subtype, in Alberta province;^17^ 40–63% in a sentinel surveillance network^18^), and Europe (14–53% depending on study and subtype);^19^ furthermore, vaccine effectiveness was highest against influenza B, consistent with early-season 2023–24 northern hemisphere influenza vaccine effectiveness estimates reported from the USA^16^ and Canada.^17^ Of note, both the 2023 southern hemisphere and 2023–24 northern hemisphere influenza vaccine formulations contained the same influenza A(H3N2) (A/Darwin/9/2021 [H3N2]-like virus) and influenza B (B/Austria/1359417/2021 [B/Victoria lineage]-like virus) components.^7^, ^20^ These findings emphasise the potential utility of southern hemisphere formulation vaccine effectiveness estimates to foreshadow, prepare for, and communicate about the following northern hemisphere season.

Among the two countries with sufficient data to calculate vaccine effectiveness against ICU admission (Australia and Chile), estimates of protection were 67·7–69·7%; previous analyses across multiple settings and seasons have demonstrated attenuation of severe influenza illness as a result of vaccination.^21^, ^22^, ^23^, ^24^ Furthermore, among the vaccination target groups evaluated, vaccine effectiveness against hospitalisation was highest among young children aged 1–4 years compared with both people aged 5–64 years with underlying health conditions and older adults aged 65 years or older. This finding might reflect reduced immunogenicity among older adults and people with underlying health conditions and is consistent with previous literature indicating lower vaccine effectiveness in older adults compared with younger age groups.^25^ In some settings, higher dose and adjuvanted influenza vaccines are recommended for use among older adults,^26^ but among the countries included in this analysis, they were only available for use in Argentina, Australia, and New Zealand, which could have contributed to the higher vaccine effectiveness estimates observed in these countries.

As in a previous analysis evaluating multicountry southern hemisphere influenza vaccine effectiveness,^6^ we observed considerable heterogeneity in influenza virus circulation and resulting vaccine effectiveness estimates across countries. For example, 2023 influenza detections in New Zealand and Thailand did not align with the typical May–July peak expected in southern hemisphere countries, and whereas influenza A(H3N2) was detected in specimens from Australia, New Zealand, and Thailand, it was not detected among specimens from REVELAC-i. Although sequencing data were not available from all countries, circulating clades probably varied across countries. Additionally, countries varied in other characteristics, such as age structure of the underlying population, national influenza vaccination policies, vaccine products used, vaccination coverage, and health-care-seeking behaviours, which probably further contributed to the observed heterogeneity. Furthermore, among the included countries, influenza vaccination coverage was frequently low and ranged broadly from 6% to 59%, with even greater variation within target groups, highlighting the importance of improving influenza vaccination coverage globally. Nonetheless, heterogeneity is to be expected and even neighbouring countries can have differences in influenza circulation;^6^, ^27^ multiregion estimates have value in providing a global view of the vaccine's performance during the influenza season.

These findings are subject to several notable limitations. First, although one of the strengths of this analysis was that we pooled data from multiple vaccine effectiveness networks using a test-negative design, there were still differences in study design and case or exposure ascertainment across sites; for example, Australian hospitalisation surveillance used a modified SARI case definition. Furthermore, not all countries were able to validate vaccination status for all patients based on a vaccination registry; for example, vaccination status in New Zealand was largely based on self-report among patients who consented to being interviewed. These methodological differences could have provided another source of heterogeneity in addition to the characteristics mentioned previously, and thus any pooled estimates we generated must be interpreted in the context of the individual country-specific and network-specific estimates. Second, as described in the previous multicountry analysis,^6^ the sensitivity of statistical tests to detect heterogeneity is limited. Third, we evaluated vaccine effectiveness from any 2023 southern hemisphere influenza vaccine and could not generate product-specific (eg, quadrivalent *vs* trivalent) or brand-specific estimates because these data were not recorded at the individual patient level for all participating countries; however, vaccine effectiveness is expected to be similar for quadrivalent and trivalent vaccines, given the absence of B/Yamagata circulation since 2020.^28^ Fourth, we stratified vaccine effectiveness estimates for a standard set of vaccination target groups (young children aged 1–4 years, children and adults aged 5–64 years with underlying health conditions, and older adults aged ≥65 years), which might not reflect vaccination policies in all countries. We restricted the youngest age group to children older than 1 year, rather than 6 months, as a conservative approach to allow for receipt of two vaccine doses before illness onset; however, it is possible that some children were partially immunised, having received only one dose. Fifth, we could not evaluate vaccine effectiveness for additional priority groups (eg, pregnant people or health-care workers) because of incompleteness or absence of such indicator variables. Finally, of the original 31 368 patients admitted with SARI across all sites, 13 109 (41·8%) did not meet eligibility criteria and 5653 (18·0%) did not have complete information on vaccination status or covariates. Although our exclusions were applied to reduce the opportunity for misclassification and other biases, it is possible that the degree of missingness of data could have resulted in additional uncontrolled bias. The completeness of data in these systems has improved over time and as it continues to do so, missing data as a source of bias will become less problematic. Similarly, sufficient data to evaluate vaccine effectiveness against ICU admission were only available from two countries, limiting the pooling and generalisability of results.

In conclusion, because of long-standing country and regional efforts to use influenza surveillance systems to routinely evaluate seasonal vaccine effectiveness, we were able to provide estimates of the effectiveness of the 2023 southern hemisphere vaccine against hospitalisation across multiple regions. Timely, pooled, end-of-season southern hemisphere vaccine effectiveness estimates have utility for multiple purposes: guiding mid-season northern hemisphere risk communication; informing February influenza vaccine composition deliberations; and allowing for evaluation of outcomes limited by sample size. Future modernisation of surveillance systems, such as initiatives to link epidemiological, molecular, genomic, and vaccination data at sentinel sites, could have even greater utility in yielding vaccine effectiveness estimates for public health practice.

### Contributors

### Data sharing

The data in this study were collected from existing national surveillance systems. The authors do not have permission to share individual-level data for other purposes. However, Stata and R scripts used to conduct the analysis could be provided upon reasonable request made to the corresponding author.

## Declaration of interests

AKR reports grants from the National Institutes of Health (USA) and membership of a data safety monitoring board for Moderna TX. ACC reports grants from the National Health and Medical Research Council, Australian Department of Health and Aged Care, and is a member of the Australian Technical Advisory Group on Immunization, which advises the Australian Minister for Health on immunisation issues. SGS reports grants from the National Institutes of Health (USA), National Health and Medical Research Council (Australia), and Department of Foreign Affairs and Trade, Australia, as well as consulting fees from Pfizer, CSL Sequiris, Moderna, Evo Health, and Novavax. All other authors declare no competing interests.
