# Supplementary material for: Effectiveness of 2023 southern hemisphere influenza vaccines against severe influenza-associated illness: pooled estimates from eight countries using the test-negative design
Source: Lancet Glob Health. 2025 Jan 29;13(2):e203–11. doi: 10.1016/S2214-109X(24)00473-X (PMC11783037; doi:10.1016/S2214-109X(24)00473-X)
Supplement: Supplementary appendix [file mmc1.pdf]

THE LANCET
Global Health

Supplementary appendix

This appendix formed part of the original submission and has been peer reviewed. We post it as supplied by the authors.

Supplement to: Gharpure R, Regan AK, Nogareda F, et al. Effectiveness of 2023 southern hemisphere influenza vaccines against severe influenza-associated illness: pooled estimates from eight countries using the test-negative design. *Lancet Glob Health* 2025; **13:** e203–11.

Supplemental Material to: Gharpure, Regan et al. Effectiveness of 2023 Southern Hemisphere influenza vaccines against severe influenza-associated illness – pooled estimates from eight countries using the test-negative design.

Table of Contents

Table S1. Summary of methods for Southern Hemisphere influenza vaccine effectiveness estimation – eight countries, 2023……………………………………………………………………………………………………3

Figure S1. Study sample and exclusions applied for Southern Hemisphere influenza vaccine effectiveness estimation – eight countries, 2023…………………………………………………………………………..…6

Table S2. Estimated Southern Hemisphere influenza vaccine effectiveness against severe acute respiratory infection (SARI) hospitalization – eight countries, 2023………………………………………………………………...7

Table S3. Estimated Southern Hemisphere influenza vaccine effectiveness against intensive care unit (ICU) admission – eight countries, 2023…………………………………………………………………………...…9

Table S4. Estimated Southern Hemisphere influenza vaccine effectiveness against severe acute respiratory infection (SARI) hospitalization among children <5 years old – eight countries, 2023……………………...………….....…11

Figure S2. Estimated Southern Hemisphere influenza vaccine effectiveness against severe acute respiratory infection (SARI) hospitalization, by virus type and subtype, high-risk group and country – eight countries, 2023……………………………………………………………………………………………………...13

Table S5. Estimated Southern Hemisphere influenza vaccine effectiveness against severe acute respiratory infection (SARI) hospitalization among persons 5-64 years old with an underlying health condition – eight countries, 2023……………………………………………………………………………………………………...15

Table S6. Estimated Southern Hemisphere influenza vaccine effectiveness against severe acute respiratory infection (SARI) hospitalization among adults ≥65 years old – eight countries, 2023…………………………………………17

Table S1. Summary of methods for Southern Hemisphere influenza vaccine effectiveness estimation – eight countries, 2023.

Characteristic	Argentina, Brazil, Chile, Paraguay, Uruguay (REVELAC-i)	Australia	New Zealand	Thailand
**Source populations**	486 sentinel hospitals in Argentina (11), Brazil (455), Chile (8), Paraguay (2), and Uruguay (10)	21 sentinel hospitals	4 sentinel hospitals	9 sentinel hospitals
**Clinical case definition[1]**	SARI: history of fever or documented temperature of ≥38°C and cough, with onset during the preceding 10 days, resulting in hospitalization	Modified SARI: Patient admitted to hospital who<br><br>a. Presents with an acute respiratory infection (>16 years: acute respiratory symptoms; children <16 years acute respiratory symptoms OR fever)<br><br>AND<br><br>b. tests positive on influenza nucleic acid testing (NAT) who are admitted to hospital	SARI: history of fever or documented temperature of ≥38°C and cough, with onset during the preceding 10 days, resulting in hospitalization	SARI: history of fever or documented temperature of ≥38°C and cough, with onset during the preceding 10 days, resulting in hospitalization
**Virological testing**	RT-PCR testing performed in National Influenza Centers and national laboratory networks	RT-PCR testing performed at each hospital	RT-PCR testing performed by National Influenza Center, Wellington, and local hospital laboratory.	RT-PCR testing performed by Thailand National Influenza Center, Bangkok
**Cases/controls for VE estimation**	Influenza test-positive cases vs test-negative controls	Influenza test-positive cases vs. the next admitted test-negative patient (≤ 2 weeks)	Influenza test-positive cases vs test-negative controls	Influenza test-positive cases vs test-negative controls
**Vaccination status ascertainment**	Vaccination registry	Medical record, self-report, or Australian Immunisation Register (AIR)	Vaccination registry or self-report	Vaccination registry
**Vaccination coverage among influenza-negative VE controls**	Overall: 38%<br><br>Children: 34%<br><br>Older adults: 44% Persons with underlying medical conditions: 41%	Overall: 35%<br><br>Children: 15%<br><br>Older adults: 64%<br><br>Persons with underlying medical conditions: 44%	Overall: 29% Children (<18): 16%<br><br>Older adults: 51%<br><br>Persons with underlying medical conditions: 37%	Overall: 6%<br><br>Children: 3%<br><br>Older adults: 22% Persons with underlying medical conditions: 18%
**Vaccines used in 2023 season (brand, QIV/TIV)[2]**	QIV: Fluarix Quad (Paraguay), Green Cross (Paraguay)	QIV: Afluria, Fluarix Tetra, FluQuadri, Vaxigrip Tetra, Fluad Quad (for older adults), Flucelvax Quad,	QIV: Afluria Quad, Afluria Quad Junior, Fluad Quad, Sanofi FluQuadri	QIV: Fluarix Tetra, FluQuadri Tetra, Vaxigrip tetra

	TIV: Viraflu (Argentina), Influvac (Argentina, Chile), Fluxvir (for older adults, Argentina), Butantan (Brazil, Uruguay), Green Cross (Uruguay)	Fluzone High Dose, Influvac Tetra		TIV: Influvac
**Date of 2023 vaccination campaign start**	Argentina: mid-March<br><br>Brazil: mid-March to beginning of April<br><br>Chile: mid-March<br><br>Paraguay: mid- April<br><br>Uruguay: mid-April	Beginning of April (national program)	End of March	Beginning of May
**Target groups for vaccination**	Children: Argentina = 6 months to 2 years; Paraguay = 6 months to 3 years; Chile and Uruguay = 6 months to <5 years; and Brazil = 6 months to <6 years<br><br>Older adults: ≥ 60 years (Brazil and Paraguay) and ≥65 years (Argentina, Chile, and Uruguay)<br><br>Persons with underlying medical conditions (see below)	Eligibility for national program: children aged 6 months to less than 5 years; all Aboriginal and Torres Strait Islander people aged 6 months and over; people aged 6 months and over with certain medical conditions that increase their chance of severe influenza and its complications (see below); pregnant women (at any stage during pregnancy); people aged 65 years and over.[2]	All people≥65 years; Maori/Pacific persons 55–64 years; Children 6 months to 12 years; People <65 years with underlying conditions (see below); Pregnant women	Older adults: ≥65 years; Children 6 months to 2 years;<br><br>Pregnant women;<br><br>Health care workers;<br><br>Persons with underlying medical conditions (see below)
**Underlying medical conditions included**	Asthma, cancer, hypertension, diabetes, cardiovascular disease, respiratory disease (excluding asthma), obesity, and immunocompromising conditions	Cardiac disease, chronic respiratory condition, immunocompromising condition, diabetes, chronic neurological condition, chronic liver disease, obesity, chronic renal disease	Diabetes, asthma, chronic obstructive pulmonary disease, other chronic lung disease , tuberculosis, cardiac disease, chronic neurological disease, current alcohol/drug dependency, active cancer, immunocompromised, chronic hematologic disorder, chronic renal disease, chronic liver disease, hypertension, other chronic disease	Chronic obstructive pulmonary disease, asthma, heart diseases, stroke, kidney failure, cancer that requires chemotherapy, diabetes, thalassemia, obesity, immunodeficiency (including HIV/AIDS)
**Period of influenza virus season[4]**	Argentina: Apr 23 to Aug 12<br><br>Brazil: Apr 30 to Sep 30<br><br>Chile: Mar 26 to Dec 2<br><br>Paraguay: Apr 30 to Aug 5<br><br>Uruguay: Apr 30 to Aug 26	Mar 19 to Oct 28	Mar 12 to Oct 21	May 28 to Nov 25

[1]Standardized SARI case definitions described in the World Health Organization's global epidemiologic surveillance standards for influenza (https://www.who.int/teams/global-influenza-programme/surveillance-and-monitoring/case-definitions-for-ili-and-sari).

[2]Inclusive of vaccines available through the national vaccination program and private market.

[3]Vaccination policy for the national program; some individual states and territories fund vaccination for additional target groups or offer universal influenza vaccination.

[4]Seasonality of influenza virus circulation was assessed by examining the distribution of test-positive influenza cases over time in the surveillance data and cross-referencing with FluNet surveillance data for each country (https://www.who.int/tools/flunet).

Figure S1. Study sample and exclusions applied for Southern Hemisphere influenza vaccine effectiveness estimation – eight countries, 2023.

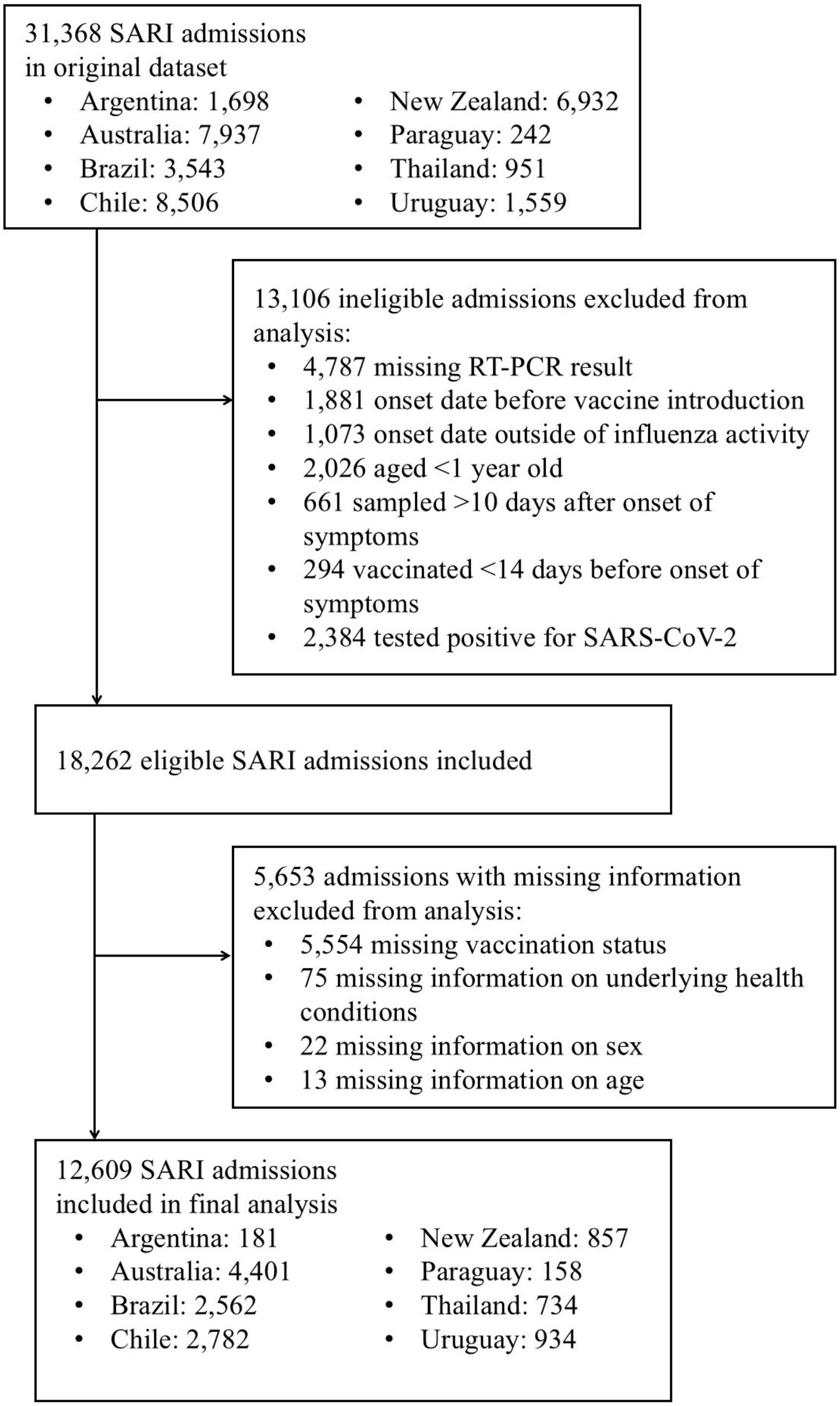

Table S2. Estimated Southern Hemisphere influenza vaccine effectiveness against severe acute respiratory infection (SARI) hospitalization – eight countries, 2023.

	**Test-Positive (TP) Cases**		**Test-Negative (TN) Controls**		**Unadjusted VE (95% CI)**	**Adjusted VE (95% CI)***
	**Vaccinated TP Cases**	**Total TP Cases**	**Vaccinated TN Controls**	**Total Controls**		
**Any influenza**						
Total	672	4,388	2,858	8,221	66·1 (62·7, 69·1)	67·2 (63·3, 70·7)
Australia	380	2,856	541	1,545	71·5 (66·8, 75·6)	67·5 (60·8, 73·0)
New Zealand	28	207	190	650	62·1 (41·0, 76·3)	58·8 (34·5, 74·1)
REVELAC-i	257	1,171	2,091	5,446	54·9 (47·5, 61·2)	43·9 (33·8, 52·5)
Argentina	4	35	23	146	NC	NC
Brazil	127	509	643	2,053	27·1 (8·6, 42·1)	29·3 (8·8, 45·1)
Chile	115	451	1,313	2,331	73·5 (66·5 79·0)	56·9 (44·8, 66·3)
Paraguay	0	44	21	114	NC	NC
Uruguay	11	132	91	802	29·0 (-38·3, 66·7)	15·5 (-70·1, 58·1)
Thailand	7	154	36	580	28·0 (-68·4, 73·5)	38·8 (-49·1, 74·9)
**Influenza A**						
Total	567	3,072	2,858	8,221	57·5 (52·9, 61·7)	62·8 (58·0, 67·0)
Australia	324	1,824	541	1,545	59·9 (52·8, 65·9)	59·2 (50·5, 66·3)
New Zealand	24	147	190	650	52·8 (23·5, 71·7)	56·3 (27·7, 73·5)
REVELAC-i	213	980	2,091	5,446	55·4 (47·5, 62·2)	43·1 (31·8, 52·5)
Argentina	4	35	23	146	NC	NC
Brazil	106	409	643	2,053	23·3 (2·0, 40·3)	25·4 (1·7, 43·4)
Chile	94	386	1,313	2,331	75·0 (67·9, 80·7)	55·6 (41·8, 66·1)
Paraguay	0	40	21	114	NC	NC
Uruguay	9	110	91	802	30·4 (-43·9, 70·1)	21·5 (-69·8, 63·7)
Thailand	6	121	36	580	21·2 (-94·9, 73·5)	39·4 (-56·5, 76·5)
**Influenza A(H1N1)pdm09**						
Total	349	2,022	2,858	8,221	60·9 (55·6, 65·5)	65·0 (59·7, 69·7)
Australia	181	1,137	541	1,545	64·9 (57·3, 71·1)	59·8 (49·7, 67·8)
New Zealand	5	35	190	650	59·6 (-7·3, 87·9)	NR
REVELAC-i	159	771	2,091	5,446	58·3 (49·8, 65·4)	43·1 (31·8, 52·5)
Argentina	0	14	23	146	NC	NC
Brazil	57	233	643	2,053	29·0 (2·2, 49·0)	31·0 (1·4, 51·8)
Chile	93	381	1,313	2,331	75·0 (67·8, 80·7)	55·4 (41·4, 66·0)
Paraguay	0	40	21	114	NC	NC
Uruguay	9	103	91	802	25·2 (-55·0, 67·9)	18·1 (-77·5, 62·2)
Thailand	4	79	36	580	NC	NC
**Influenza A(H3N2)**						
Total	26	167	2,858	8,221	65·4 (47·0, 78·2)	67·2 (45·9, 80·1)
Australia	19	114	541	1,545	62·9 (38·0, 78·8)	71·5 (47·7, 84·4)
New Zealand	5	14	190	650	NR	NR
REVELAC-i	0	0	2,091	5,446	NC	NC
Argentina	0	0	23	146	NC	NC
Brazil	0	0	643	2,053	NC	NC
Chile	0	0	1,313	2,331	NC	NC
Paraguay	0	0	21	114	NC	NC
Uruguay	0	0	91	802	NC	NC
Thailand	2	39	36	580	NC	NC
**Influenza B**						
Total	105	1,305	2,858	8,221	83·6 (79·8, 86·7)	78·0 (72·2, 82·6)
Australia	56	1,020	541	1,545	89·2 (85·6, 92·1)	84·7 (78·3, 89·2)
New Zealand	4	60	190	650	NC	NC
REVELAC-i	44	191	2,091	5,446	52·0 (32·0, 66·7)	43·1 (31·8, 52·5)

Argentina	0	0	23	146	NC	NC
Brazil	21	100	643	2,053	41·7 (3·7, 66·1)	37·5 (-4·2, 62·5)
Chile	21	65	1,313	2,331	63·0 (35·9, 79·2)	64·5 (37·9, 79·7)
Paraguay	0	4	21	114	NC	NC
Uruguay	2	22	91	802	NC	NC
Thailand	1	34	36	580	NC	NC

Abbreviations: CI, confidence interval; NC, not calculated (insufficient sample size); NR, not reported (due to confidence interval); TN, test negative; TP, test positive; VE, vaccine effectiveness.

*Adjusted for age group (1–4 years, 5–64 years, and ≥65 years), sex, underlying health conditions, and week of illness onset (fit as cubic spline).

Table S3. Estimated Southern Hemisphere influenza vaccine effectiveness against intensive care unit (ICU) admission – eight countries, 2023.

	**Test-Positive (TP) Cases**		**Test-Negative (TN) Controls**		**Unadjusted VE (95% CI)**	**Adjusted VE. (95% CI)***
	**Vaccinated TP Cases**	**Total TP Cases**	**Vaccinated TN Controls**	**Total TN Controls**		
**Any influenza**						
Total	73	443	370	955	68·8 (58·3, 76·8)	67·7 (55·0, 76·9)
Australia	39	253	55	132	74·5 (57·3, 84·8)	69·7 (45·3, 83·3)
New Zealand	5	27	18	60	NR	NR
REVELAC-i	28	156	293	714	68·6 (50·9, 80·4)	57·4 (30·9, 73·7)
Argentina	2	8	4	23	NC	NC
Brazil	--	--	--	--	NC	NC
Chile	21	114	260	512	78·1 (63·2, 87·4)	67·7 (44·5, 81·2)
Paraguay	0	5	2	10	NC	NC
Uruguay	5	29	27	169	NR	NR
Thailand	1	7	4	49	NC	NC
**Influenza A**						
Total	58	318	370	955	64·7 (51·4, 74·7)	64·7 (49·3, 75·4)
Australia	30	159	55	132	67·4 (43·1, 81·5)	66·2 (36·9, 81·9)
New Zealand	4	14	18	60	NC	NC
REVELAC-i	23	139	293	714	71·5 (53·8, 83·0)	57·9 (29·0, 75·0)
Argentina	2	8	4	23	NC	NC
Brazil	---	---	---	---	NC	NC
Chile	16	100	260	512	81·5 (67·1, 90·2)	70·3 (45·5, 83·8)
Paraguay	0	4	2	10	NC	NC
Uruguay	5	27	27	142	NR	NR
Thailand	1	6	4	49	NC	NC
**Influenza A(H1N1)pdm09**						
Total	37	218	370	955	67·7 (52·5, 78·4)	64·4 (46·1, 76·5)
Australia	14	81	55	132	70·7 (40·6, 86·2)	65·8 (25·6, 84·2)
New Zealand	2	6	18	60	NC	NC
REVELAC-i	21	128	293	714	71·8 (53·4, 83·6)	57·7 (26·7, 75·5)
Argentina	0	1	4	23	NC	NC
Brazil	---	---	---	---	NC	NC
Chile	16	96	260	512	80·6 (65·4, 89·7)	68·5 (42·1, 82·8)
Paraguay	0	4	2	10	NC	NC
Uruguay	5	27	27	169	NR	NR
Thailand	0	3	4	49	NC	NC
**Influenza A(H3N2)**						
Total	4	10	370	955	NC	NC
Australia	2	5	55	132	NC	NC
New Zealand	1	2	18	60	NC	NC
REVELAC-i	28	156	293	714	NC	NC
Argentina	0	0	4	23	NC	NC
Brazil	---	---	---	---	NC	NC
Chile	0	0	260	512	NC	NC
Paraguay	0	0	2	10	NC	NC
Uruguay	0	0	27	169	NC	NC
Thailand	1	3	4	49	NC	NC
**Influenza B**						
Total	15	125	370	955	78·4 (62·1, 88·5)	74·7 (50·9, 86·9)
Australia	9	94	55	132	85·2 (66·9, 93·9)	83·2 (56·2, 93·5)
New Zealand	1	13	18	60	NC	NC
REVELAC-i	5	17	293	714	NR	67·2 (-6·4, 89·9)

Argentina	0	0	4	23	NC	NC
Brazil					NC	NC
Chile	5	14	260	512	NR	66·7 (-13·3, 90·2)
Paraguay	0	1	2	10	NC	NC
Uruguay	0	2	27	169	NC	NC
Thailand	0	1	4	49	NC	NC

Abbreviations: CI, confidence interval; NC, not calculated (insufficient sample size); NR, not reported (due to confidence interval >140%); TN, test negative; TP, test positive; VE, vaccine effectiveness.

*Adjusted for age group (1–4 years, 5–64 years, and ≥65 years), sex, underlying health conditions, and week of illness onset (fit as cubic spline).

Table S4. Estimated Southern Hemisphere influenza vaccine effectiveness against severe acute respiratory infection (SARI) hospitalization among children <5 years old – eight countries, 2023.

	Test-Positive (TP) Cases		Test-Negative (TN) Controls		Unadjusted VE (95% CI)	Adjusted VE (95% CI)*
	Vaccinated TP Cases	Total TP Cases	Vaccinated TN Controls	Total TN Controls		
**Any influenza**						
Total	66	1,101	915	3,338	83·1 (78·0, 87·2)	74·7 (65·9, 81·2)
Australia	25	752	66	448	80·1 (67·4, 88·2)	87·6 (77·1, 93·3)
New Zealand	3	59	28	208	NC	NC
REVELAC-i	37	236	809	2,326	65·1 (49·7, 76·4)	56·8 (37·8, 70·1)
Argentina	0	3	7	34	NC	NC
Brazil	24	145	414	1,362	54·6 (27·9, 72·4)	46·3 (14·2, 66·4)
Chile	13	42	353	553	74·6 (48·1, 88·1)	71·3 (41·3, 85·9)
Paraguay	0	10	15	65	NC	NC
Uruguay	0	36	20	312	NC	NC
Thailand	1	54	12	356	NC	NC
**Influenza A**						
Total	51	769	915	3,338	81·2 (74·7, 86·3)	73·1 (62·5, 80·7)
Australia	22	513	66	448	74·1 (56·5, 85·0)	84·6 (70·8, 91·8)
New Zealand	3	35	28	208	NC	NC
REVELAC-i	25	176	809	2,326	69·0 (51·9, 80·7)	60·8 (39·3, 74·7)
Argentina	0	3	7	34	NC	NC
Brazil	17	105	414	1,362	55·8 (23·9, 75·6)	48·0 (10·2, 69·9)
Chile	8	32	353	553	81·1 (55·4, 92·8)	75·2 (41·2, 89·5)
Paraguay	0	8	15	65	NC	NC
Uruguay	0	28	20	312	NC	NC
Thailand	1	45	12	356	NC	NC
**Influenza A(H1N1)pdm09**						
Total	29	503	915	3,338	83·8 (76·2, 89·3)	78·0 (66·4, 85·6)
Australia	11	348	66	448	81·1 (63·1, 91·1)	87·6 (73·6, 94·2)
New Zealand	2	10	28	208	NC	NC
REVELAC-i	15	110	809	2,326	70·4 (48·2, 84·2)	64·9 (37·2, 80·4)
Argentina	0	3	7	34	NC	NC
Brazil	7	44	414	1,362	56·7 (0·5, 83·8)	48·8 (-18·0, 77·8)
Chile	8	32	353	553	81·1 (55·4, 92·8)	75·2 (41·2, 89·5)
Paraguay	0	8	15	65	NC	NC
Uruguay	0	23	20	312	NC	NC
Thailand	1	35	12	356	NC	NC
**Influenza A(H3N2)**						
Total	4	49	915	3,338	NC	NC
Australia	4	37	66	448	NC	NC
New Zealand	0	2	28	208	NC	NC
REVELAC-i	0	0	809	2,326	NC	NC
Argentina	0	0	7	34	NC	NC
Brazil	0	0	414	1,362	NC	NC
Chile	0	0	353	553	NC	NC
Paraguay	0	0	15	65	NC	NC
Uruguay	0	0	20	312	NC	NR
Thailand	0	10	12	356	NC	NC
**Influenza B**						
Total	15	329	915	3,338	87·3 (78·7, 93·0)	82·1 (68·4, 89·8)
Australia	3	236	66	448	NC	NC
New Zealand	0	24	28	208	NC	NC
REVELAC-i	12	60	809	2,326	53·1 (9·8, 77·5)	46·8 (-1·4, 72·1)

Argentina	0	0	7	34	NC	NC
Brazil	7	40	414	1,362	51·4 (-12·9, 82·0)	42·0 (-34·9, 75·0)
Chile	5	10	353	553	NR	NR
Paraguay	0	2	15	65	NC	NC
Uruguay	0	8	20	312	NC	NC
Thailand	0	9	12	356	NC	NC

Abbreviations: CI, confidence interval; NC, not calculated (insufficient sample size); NR, not reported (due to confidence interval); TN, test negative; TP, test positive; VE, vaccine effectiveness.

*Adjusted for age group (1–4 years, 5–64 years, and ≥65 years), sex, underlying health conditions, and week of illness onset (fit as cubic spline).

Figure S2. Estimated Southern Hemisphere influenza vaccine effectiveness against severe acute respiratory infection (SARI) hospitalization, by virus type and subtype, high-risk group and country – eight countries,* 2023.

Any Influenza

Target Group, Country	TP Cases Vaccinated	TP Cases Total	TN Controls Vaccinated	TN Controls Total	VE	[95% CI]
**Children (<5 years)**						
Australia	25	752	66	448	87.6	[77.1; 93.3]
Brazil	24	145	414	1362	46.3	[14.2; 66.4]
Chile	13	42	353	553	71.3	[41.3; 85.9]
**Random effects model**					**70.9**	**[47.5; 94.4]**
Heterogeneity: I^2 = 80%, τ^2 = 333.61, p < 0.01						
**≥1 health condition (5-64 years)**						
Australia	172	980	124	447	59.3	[45.9; 69.4]
Chile	27	148	227	511	50.3	[18.8; 69.6]
New Zealand	13	64	60	204	28.2	[-44.9; 64.4]
**Random effects model**					**56.6**	**[46.2; 67.1]**
Heterogeneity: I^2 = <1%, τ^2 = 0, p = 0.48						
**Older adults (≥65 years)**						
Australia	165	342	336	525	59.3	[45.0; 69.8]
Brazil	98	313	181	486	14.4	[-19.1; 38.5]
Chile	58	167	496	826	39.9	[11.7; 59.1]
New Zealand	9	37	90	178	72.2	[34.7; 88.2]
**Random effects model**					**47.7**	**[24.9; 70.5]**
Heterogeneity: I^2 = 73%, τ^2 = 403.22, p = 0.01						

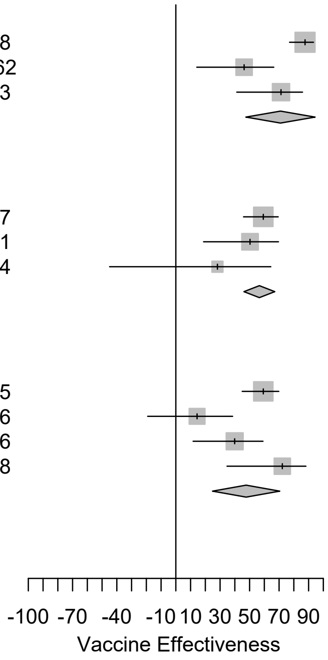

Influenza A

Target group, Country	TP Cases Vaccinated	TP Cases Total	TN Controls Vaccinated	TN Controls Total	VE	[95% CI]
**Children (<5 years)**						
Australia	22	513	66	448	84.6	[70.8; 91.8]
Brazil	17	105	414	1362	48.0	[10.2; 69.9]
Chile	8	32	353	553	75.2	[41.2; 89.5]
**Random effects model**					**73.0**	**[53.2; 92.8]**
Heterogeneity: I^2 = 62%, τ^2 = 192.50, p = 0.07						
**≥1 health condition (5-64 years)**						
Australia	134	599	124	447	42.8	[22.6; 57.8]
Chile	23	126	227	511	45.0	[5.4; 68.1]
New Zealand	11	49	60	204	14.4	[-84.7; 60.3]
**Random effects model**					**42.1**	**[27.1; 57.1]**
Heterogeneity: I^2 = 0%, τ^2 = 0, p = 0.74						
**Older adults (≥65 years)**						
Australia	155	315	336	525	57.7	[42.5; 68.8]
Brazil	84	267	181	486	12.0	[-24.8; 37.9]
Chile	53	161	496	826	43.5	[16.1; 61.9]
New Zealand	9	34	90	178	68.7	[25.8; 86.8]
**Random effects model**					**47.0**	**[26.0; 68.0]**
Heterogeneity: I^2 = 65%, τ^2 = 305.14, p = 0.03						

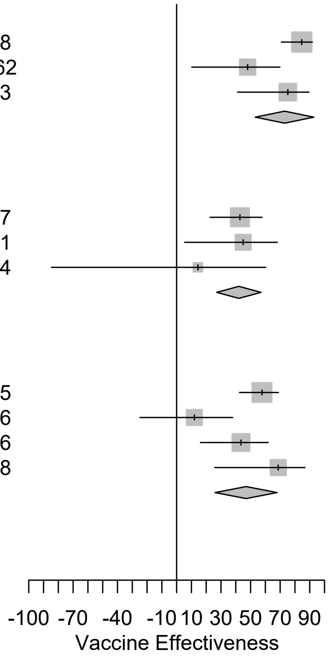

Figure S2 (cont'd). Estimated Southern Hemisphere influenza vaccine effectiveness against severe acute respiratory infection (SARI) hospitalization, by virus type and subtype, high-risk group and country – eight countries,* 2023

Influenza A(H1N1)pdm09

Target group, Country	TP Cases Vaccinated	TP Cases Total	TN Controls Vaccinated	TN Controls Total	VE	[95% CI]
**Children (<5 years)**						
Australia	11	348	66	448	87.6	[73.6; 94.2]
Brazil	7	44	414	1362	48.8	[-18.0; 77.8]
Chile	8	32	353	553	75.2	[41.2; 89.5]
**Random effects model**					**80.9**	**[66.1; 95.7]**
Heterogeneity: I^2 = 34%, τ^2 = 60.4009, p = 0.22						
**≥1 health condition (5-64 years)**						
Australia	72	351	124	447	42.6	[18.3; 59.6]
Chile	23	125	227	511	45.0	[5.3; 68.0]
**Older adults (≥65 years)**						
Australia	91	185	336	525	55.5	[36.3; 69.0]
Brazil	49	171	181	486	21.6	[-18.6; 48.1]
Chile	52	159	496	826	44.1	[16.8; 62.5]
**Random effects model**					**45.2**	**[28.5; 61.8]**
Heterogeneity: I^2 = 40%, τ^2 = 81.9467, p = 0.19						

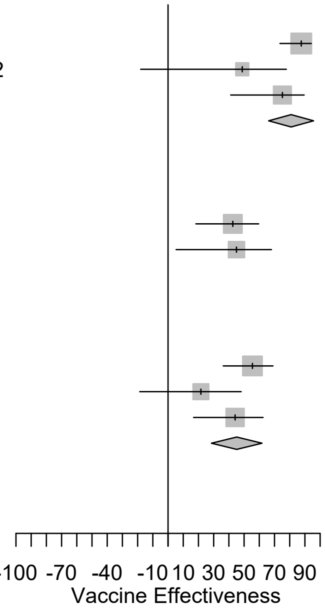

Influenza B

Target group, Country	TP Cases Vaccinated	TP Cases Total	TN Controls Vaccinated	TN Controls Total	VE	[95% CI]
**Children (<5 years)**						
Brazil	7	40	414	1362	42.0	[-34.9; 75.0]
**≥1 health condition (5-64 years)**						
Australia	38	375	124	447	78.5	[67.3; 85.9]
**Older adults (≥65 years)**						
Australia	10	27	336	525	70.9	[33.4; 87.3]
Brazil	14	46	181	486	21.2	[-55.8; 60.1]

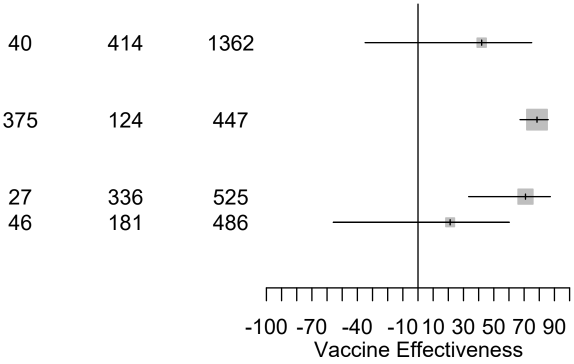

Abbreviations: CI, confidence interval; TN, test negative; TP, test positive; VE, vaccine effectiveness.

*Estimates are shown only for countries with sufficient data. Estimates are adjusted for age group (1–4 years, 5–64 years, and ≥65 years), sex, underlying health conditions, and week of illness onset (fit as cubic spline).

Table S5. Estimated Southern Hemisphere influenza vaccine effectiveness against severe acute respiratory infection (SARI) hospitalization among persons 5-64 years old with an underlying health condition – eight countries, 2023.

	Test-Positive (TP) Cases		Test-Negative (TN) Controls		Unadjusted VE (95% CI)	Adjusted VE (95% CI)*
	Vaccinated TP Cases	Total TP Cases	Vaccinated TN Controls	Total TN Controls		
**Any influenza**						
Total	217	1,234	430	1,330	55·3 (46·0, 63·1)	53·4 (42·2, 62·4)
Australia	172	980	124	447	44·6 (27·0, 57·8)	59·3 (45·9, 69·4)
New Zealand	13	64	60	204	38·8 (-24·7, 71·6)	28·2 (-44·9, 64·4)
REVELAC-i	31	172	241	640	63·6 (43·9, 76·9)	39·8 (3·6, 62·4)
Argentina	0	0	0	7	NC	NC
Brazil	0	0	0	0	NC	NC
Chile	27	148	227	511	72·1 (55·5, 82·9)	50·3 (18·8, 69·6)
Paraguay	0	0	0	0	NC	NC
Uruguay	4	23	14	122	NC	NC
Thailand	1	18	5	39	NC	NC
**Influenza A**						
Total	172	811	430	1,330	43·7 (30·6, 54·3)	39·8 (24·,3 52·1)
Australia	134	599	124	447	24·9 (-0·6, 44·0)	42·8 (22·6, 57·8)
New Zealand	11	49	60	204	30·5 (-50·5, 70·0)	14·4 (-84·7, 60·3)
REVELAC-i	26	147	241	640	64·4 (43·3, 78·3)	34·8 (-9·9, 61·3)
Argentina	0	0	0	7	NC	NC
Brazil	0	0	0	0	NC	NC
Chile	23	126	227	511	72·1 (54·0, 83·6)	45·0 (5·4, 68·1)
Paraguay	0	0	0	0	NC	NC
Uruguay	3	20	14	122	NC	NC
Thailand	1	16	5	39	NC	NC
**Influenza A(H1N1)pdm09**						
Total	98	513	430	1,330	50·6 (36·3, 61·8)	43·4 (25·5, 57·0)
Australia	72	351	124	447	32·8 (5·2, 52·5)	42·6 (18·3, 59·6)
New Zealand	0	10	60	204	NC	NC
REVELAC-i	26	145	241	640	63·8 (42·4, 77·9)	34·4 (-10·5, 61·1)
Argentina	0	0	0	7	NC	NC
Brazil	0	0	0	0	NC	NC
Chile	23	125	227	511	71·8 (53·5, 83·4)	45·0 (5·3, 68·0)
Paraguay	0	0	0	0	NC	NC
Uruguay	3	20	14	122	NC	NC
Thailand	0	7	5	39	NC	NC
**Influenza A(H3N2)**						
Total	8	42	430	1,330	50·8 (-9·6, 80·5)	44·9 (-26·7, 76·0)
Australia	4	27	124	447	NC	NC
New Zealand	3	6	60	204	NC	NC
REVELAC-i	0	0	241	640	NC	NC
Argentina	0	0	0	7	NC	NC
Brazil	0	0	0	0	NC	NC
Chile	0	0	227	511	NC	NC
Paraguay	0	0	0	0	NC	NC
Uruguay	0	0	14	122	NC	NC
Thailand	1	9	5	39	NC	NC
**Influenza B**						
Total	45	417	430	1,330	74·7 (64·6, 82·2)	75·7 (64·7, 83·3)
Australia	38	375	124	447	70·6 (55·9, 80·7)	78·5 (67·3, 85·9)
New Zealand	2	15	60	204	NC	NC
REVELAC-i	5	25	241	640	58·6 (-15·7, 88·0)	66·6 (2·8, 88·5)

Argentina	0	0	0	7	NC	NC
Brazil	0	0	0	0	NC	NC
Chile	4	22	227	511	NC	NC
Paraguay	0	0	0	0	NC	NC
Uruguay	1	3	14	122	NC	NC
Thailand	0	2	5	39	NC	NC

Abbreviations: CI, confidence interval; NC, not calculated (insufficient sample size); NR, not reported (due to confidence interval); TN, test negative; TP, test positive; VE, vaccine effectiveness.

*Adjusted for age group (1–4 years, 5–64 years, and ≥65 years), sex, underlying health conditions, and week of illness onset (fit as cubic spline).

Table S6. Estimated Southern Hemisphere influenza vaccine effectiveness against severe acute respiratory infection (SARI) hospitalization among adults ≥65 years old – eight countries, 2023.

	Test-Positive (TP) Cases		Test-Negative (TN) Controls		Unadjusted VE (95% CI)	Adjusted VE (95% CI)*
	Vaccinated TP Cases	Total TP Cases	Vaccinated TN Controls	Total TN Controls		
**Any influenza**						
Total	342	980	1,182	2,437	43·1 (33·4, 51·3)	46·6 (36·6, 55·0)
Australia	165	342	336	525	47·6 (30·1, 60·6)	59·3 (45·0, 69·8)
New Zealand	9	37	90	178	68·6 (26·3, 87·6)	72·2 (34·7, 88·2)
REVELAC-i	166	589	746	1,688	50·4 (39·0, 59·8)	39·8 (3·6, 62·4)
Argentina	3	29	16	81	NC	NC
Brazil	98	313	181	486	23·2 (-4·9, 43·9)	14·4 (-19·1, 38·5)
Chile	58	167	496	826	64·6 (49·2, 75·4)	39·9 (11·7, 59·1)
Paraguay	0	34	6	48	NC	NC
Uruguay	7	46	47	247	NR	NR
Thailand	2	12	10	46	NC	NC
**Influenza A**						
Total	312	895	1,182	2,437	43·2 (33·2, 51·7)	47·0 (36·8, 55·6)
Australia	155	315	336	525	45·5 (26·9, 59·4)	57·7 (42·5, 68·8)
New Zealand	9	34	90	178	64·8 (16·3, 86·3)	68·7 (25·8, 86·8)
REVELAC-i	146	534	746	1,688	52·5 (40·9, 61·9)	34·8 (-9·9, 61·3)
Argentina	3	29	16	81	NC	NC
Brazil	84	267	181	486	22·7 (-7·4, 44·5)	12·0 (-24·8, 37·9)
Chile	53	161	496	826	67·3 (52·7, 77·6)	43·5 (16·1, 61·9)
Paraguay	0	32	6	48	NC	NC
Uruguay	6	45	47	247	NR	NR
Thailand	2	12	10	46	NC	NC
**Influenza A(H1N1)pdm09**						
Total	202	614	1,182	2,437	47·9 (37·0, 57·0)	44·3 (31·4, 54·7)
Australia	91	185	336	525	45·5 (22·4, 61·7)	55·5 (36·3, 69·0)
New Zealand	3	9	90	178	NC	NC
REVELAC-i	107	416	746	1,688	56·3 (44·1, 65·9)	34·4 (-10·5, 61·1)
Argentina	0	10	16	81	NC	NC
Brazil	49	171	181	486	32·3 (-0·3, 54·7)	21·6 (-18·6, 48·1)
Chile	52	159	496	826	67·7 (53·1, 77·9)	44·1 (16·8, 62·5)
Paraguay	0	32	6	48	NC	NC
Uruguay	6	44	47	247	NR	NR
Thailand	1	4	10	46	NC	NC
**Influenza A(H3N2)**						
Total	13	38	1,182	2,437	44·8 (-12·8, 74·2)	65·8 (27·7, 83·8)
Australia	10	26	336	525	64·8 (15·6, 86·0)	71·8 (33·6, 88·0)
New Zealand	2	4	90	178	NC	NC
REVELAC-i	0	0	746	1,688	NC	NC
Argentina	0	0	16	81	NC	NC
Brazil	0	0	181	486	NC	NC
Chile	0	0	496	826	NC	NC
Paraguay	0	0	6	48	NC	NC
Uruguay	0	0	47	247	NC	NC
Thailand	1	8	10	46	NC	NC
**Influenza B**						
Total	30	85	1,182	2,437	42·1 (7·3, 64·4)	41·6 (5·5, 63·9)
Australia	10	27	336	525	66·9 (21·6, 86·7)	70·9 (33·4, 87·3)
New Zealand	0	3	90	178	NC	NC
REVELAC-i	20	55	746	1,688	27·8 (-29·8, 60·9)	66·6 (2·8, 88·5)

Argentina	0	0	16	81	NC	NC
Brazil	14	46	181	486	26·3 (-46·7, 64·6)	21·2 (-55·8, 60·1)
Chile	5	6	496	826	NR	NR
Paraguay	0	2	6	48	NC	NC
Uruguay	1	1	47	247	NC	NC
Thailand	0	0	10	46	NC	NC

Abbreviations: CI, confidence interval; NC, not calculated (insufficient sample size); NR, not reported (due to confidence interval); TN, test negative; TP, test positive; VE, vaccine effectiveness.

*Adjusted for age group (1–4 years, 5–64 years, and ≥65 years), sex, underlying health conditions, and week of illness onset (fit as cubic spline).
